# Associations and correlates of general versus specific successful ageing components

**DOI:** 10.1007/s10433-020-00593-4

**Published:** 2020-12-12

**Authors:** Myriam V. Thoma, Luca Kleineidam, Simon Forstmeier, Andreas Maercker, Siegfried Weyerer, Marion Eisele, Hendrik van den Bussche, Hans-Helmut König, Susanne Röhr, Janine Stein, Birgitt Wiese, Michael Pentzek, Horst Bickel, Wolfgang Maier, Martin Scherer, Steffi G. Riedel-Heller, Michael Wagner

**Affiliations:** 1 Psychopathology and Clinical Intervention, University of Zurich, Zurich, Switzerland; 2 Dynamics of Healthy Ageing, University Research Priority Program, University of Zurich, Zurich, Switzerland; 3 Department of Neurodegenerative Diseases and Geriatric Psychiatry, University of Bonn, Bonn, Germany; 4 DZNE, German Center for Neurodegenerative Diseases, Bonn, Germany; 5 Faculty II, Developmental Psychology, University of Siegen, Adolf-Reichwein-Str. 2a, 57068 Siegen, Germany; 6 Medical Faculty Mannheim, Central Institute of Mental Health, Heidelberg University, Mannheim, Germany; 7 Department of Primary Medical Care, Center for Psychosocial Medicine, University Medical Center Hamburg-Eppendorf, Hamburg, Germany; 8 Institute of Social Medicine, Occupational Health and Public Health (ISAP), University of Leipzig, Leipzig, Germany; 9 Hannover Medical School, WG Medical Statistics and IT-Infrastructure, Institute of General Practice, Hannover, Germany; 10 Institute of General Practice (Ifam), Medical Faculty, Heinrich-Heine University Düsseldorf, Düsseldorf, Germany; 11 Department of Psychiatry and Psychotherapy, Klinikum Rechts Der Isar, Technical University of Munich, Munich, Germany

**Keywords:** Successful ageing, AgeCoDe, Associations and correlates, Health, Cognitive reserve

## Abstract

The heterogeneity in the operationalisation of successful ageing (SA) hinders a straightforward examination of SA associations and correlates, and in turn, the identification of potentially modifiable predictors of SA. It is unclear which SA associations and correlates influence all facets of the SA construct, and whether psychosocial reserve models developed in neuropathological ageing research can also be linked to SA. It was therefore the aim of this study to disentangle the effect of various previously identified SA associations and correlates on (1) a general SA factor, which represents the shared underpinnings of three SA facets, and (2) more confined, specific factors, using bifactor modelling. The associations and correlates of three recently validated SA operationalisations were compared in 2478 participants from the German AgeCoDe study, aged 75 years and above. Based on participants’ main occupation, cognitive reserve (CR) and motivational reserve (MR) models were built. Younger age, male gender, more education, higher socio-economic status, being married or widowed, as well as more physical exercise and cognitive activities in old age were found to correlate positively with the general SA factor, indicating a simultaneous effect on all aspects of SA. Smoking and ApoE-ε4 were related only to the physiological facet of SA. CR models were significantly related to the general SA factor. Among all SA associations and correlates, proxy indicators of lifelong cognitive activity and physical exercise showed the strongest effects on SA. Future intervention studies should assess the influence of the preservation of active lifestyle across the life span on SA.

**Electronic supplementary material:**

The online version of this article (10.1007/s10433-020-00593-4) contains supplementary material, which is available to authorised users.

## Introduction

Salutogenic ageing models, such as *active ageing,* or *successful ageing* (SA) have evolved in recent decades to counter-balance the predominantly deficit-oriented, (psycho)pathological approach to ageing. Active ageing is defined by the World Health Organization (WHO), as “… the process of optimizing opportunities for health, participation and security in order to enhance quality of life as people age” (WHO, p. 12). It is currently predominantly used in Europe as a policy framework to address population ageing (with a focus on activity, participation, and productivity of older members of society). SA has been globally established as a frequently used construct in the investigation of ageing well (e.g. Foster and Walker 2015). By today, there exist various definitions of SA (see Martin et al. 2015). The early influential psychological SA model from Baltes and Baltes (1990) understands successful lifelong development (including ageing) as a process that consists of the components selection, optimization, and compensation. One of the most influential (but also often criticized) health-based SA models is the multidimensional MacArthur model, which encompasses the lack of (risk factors for) physical disease, high cognitive and physical functioning, as well as engagement in social and productive activities (1997). However, only about 12% to 19% of older adults have been shown to fulfil these requirements (McLaughlin et al. 2010; Strawbridge et al. 2002). Due to the high personal burden, but also the societal costs associated with “non-successful” ageing trajectories, it is of extreme importance to increase these numbers. As such, there has been a growing investment in research efforts to identify associations, correlates, and predictors of SA.

Previous studies have identified predictors of SA, including younger age, race (White), weight and height, non-smoking status, physical activity, diet, as well as factors promoting favourable health behaviours, such as socio-economic status (SES: income, wealth), and education (e.g. Hodge et al. 2013). All these studies applied SA models that placed an emphasis on the physiological health and physical functioning facets of SA. Other research examined SA definitions by focusing on the individual’s subjective experience of the ageing process. These findings identified associations and correlates of SA, which included social connectedness (e.g. close friends, visits with family), resilience, functioning in everyday life, and health-related quality of life (Montross et al. 2006). Associations and correlates of SA may therefore differ as a function of the emphasis of the applied SA construct (McLaughlin et al. 2010; Pruchno et al. 2010a, b). This observation is corroborated by the findings of studies using mixed operational definitions of SA, i.e. using both objective and subjective SA criteria, which result in the identification of a broader set of SA associations and correlates, encompassing physical, social, and psychological factors (Ng et al. 2009; Vaillant and Mukamal, 2001). However, a systematic review by Cosco and colleagues identified a high number of 105 different quantitative operational SA definitions in the literature (Cosco et al. 2014). Coupled with the tentative conclusion that SA correlates may (systematically) differ as a function of the emphasis of the applied SA operationalisation, little confidence can be placed in what is currently known about SA associations, correlates, and predictors.

**Fig. 1:**
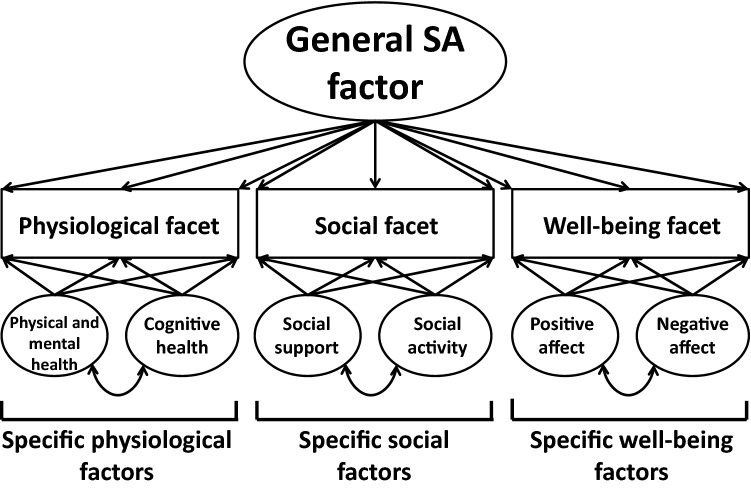
Schematic illustration of the bifactor model for successful ageing

To increase this confidence in our understanding of meaningful predictors of SA, it is important to identify factors that influence all facets of SA (both subjective and objective) in the same manner, and which may therefore be more independent from the specific operational definition applied. The identification of modifiable associations and correlates of the individual components of SA, such as physiological health or well-being, is an important research goal. However, the identification of factors that influence multiple SA facets simultaneously would be of increased relevance in promoting a favourable ageing course, as interventions targeting these variables would exert a broader effect on all facets of SA. It was therefore the first aim of this study to identify these associations and correlates of SA, using a structural equation modelling framework that was previously developed for the operationalisation of SA in the *German Study on Ageing, Cognition, and Dementia* (AgeCoDe, Kleineidam et al. 2018). In the AgeCoDe study, using bifactor models (Reise 2012), results demonstrated that a SA construct should constitute a general SA factor, which forms a latent construct representing aspects of SA shared by all commonly used SA definitions (Cosco et al. 2014); but also constitutes specific factors representing components unique to the individual SA facets. Study findings further showed that an integration of physiological, well-being, and social aspects is necessary to provide a well-balanced definition of SA (see Kleineidam et al. 2018). Therefore, the current study intends to examine whether the associations and correlates of SA proposed in the literature are related to all three of these facets via the general factor of the SA bifactor model, representing their shared underpinning of all SA facets; or whether they are selectively related to the unique factors of the model, representing more confined, facet-specific aspects of SA (see Fig. 1 for a proposed model of SA facets and related factors). Associations and correlates relating to the general factor would influence all facets of SA simultaneously and might therefore indicate important targets for public health policies. Whereas associations and correlates of the unique factors may be highly relevant for the specific health aspects, rather than SA in general. It is expected that previously proposed and well-established SA determinates, such as age, gender, education, SES, marital status, physical activity, and smoking status will be predominantly related to the general SA factor.

The second aim of this study was to broaden the set of associations and correlates typically considered in SA research by additionally examining novel factors that have been identified in neuropathological ageing research: the *apolipoprotein E* (*ApoE*) ε4 allele, and the concepts of cognitive reserve (CR), and motivational reserve (MR). The *ApoE*-ε4 allele is the strongest genetic risk factor for Alzheimer’s disease, as well as atherosclerosis, and longevity (for a review see Van Giau et al. 2015). Despite its pleiotropic effects in old age, it has not yet been investigated whether *ApoE-ε4* is also related to SA. However, it would be of great scientific relevance to ascertain whether a biological index that is already applied in the field of pathology could also be used in the prediction of salutogenic outcomes. Given that SA a) (strongly) builds on physical health, and b) is often predicted by younger age (e.g. Depp and Jeste 2006; Ng et al. 2009), it is therefore expected that the *APOE-ε4* allele, as a biological predictor, will be negatively associated with the physiological SA facet, but not associated with the social or well-being SA facets.

The concept of CR has gained a lot of interest and momentum in dementia research. The concept assumes that engagement in cognitively stimulating activities across the life span, such as educational and occupational attainment, and social and leisure activities, result in an enhanced ability to compensate pathological brain changes in later life (Barulli and Stern 2013; Forstmeier and Maercker 2008; Simon Forstmeier et al. 2012; Stern 2012). In the literature, the concept of CR has often been operationalised in terms of early-life cognitive stimulation, such as level of education. However, previous research has shown that midlife cognitive activities may be even more important for CR in later life (Reed et al. 2011). Taking this into consideration, the CR construct was examined in the current study using an index that also encompassed midlife cognitive stimulation, namely occupation-related cognitive activities. This was derived from the participant’s main (longest) occupation, i.e. job title, duration of occupation, and major activities and duties of occupation. Factors identified in previous research as relevant for increasing CR (Barulli and Stern 2013; Forstmeier and Maercker 2008; Simon Forstmeier et al. 2012; Stern 2012), have shown vast similarities to identified predictors of SA (e.g. education, SES, physical activity). Thus, it is expected that CR, which has traditionally been examined in the context of (age-related) neuropathology, may also be used to predict SA. It is therefore expected that CR will be positively related to the general SA factor.

As motivational abilities, such as for example goal orientation, self-efficacy, or action planning have received increasing attention in both basic (e.g. Boyle et al. 2013; Jimura et al. 2011) and applied (Heart and Kalderon 2013; Wahl et al. 2013) gerontology research, it is essential to include the investigation of the role of motivational abilities within the context of clinical geropsychology. In order to achieve this objective, and also to complement the CR concept, the research group of Forstmeier and Maercker developed the motivational reserve (MR) concept (Forstmeier and Maercker 2008). While the CR concept refers to specific activities that have the potential to increase brain/cognitive reserve (e.g. physical and cognitive activity), the MR concept places stronger emphasis on the motivational abilities that are required for or are related to the pursuit of such activities (e.g. the determination to achieve a particular goal). Several approaches to measure MR have been proposed, including occupation-related motivational abilities, behavioural tasks (delay of gratification), scenario tests, self-report, and informant-report measures (Forstmeier and Maercker 2015; Forstmeier et al. 2021). Since the present longitudinal study includes a high number of medical and psychological variables, only one of these approaches could be applied. The occupation-related MR measure has been chosen because it has been shown to predict risk of MCI and–in genetically predisposed individuals–AD (Forstmeier et al. 2012). It was derived from occupation-related motivational activities of the participant’s main (longest) occupation, encompassing goal orientation and action planning. Similar to the CR model, the MR model assumes a protective influence against neuropathological decline, in the context of age-related and/or pathological cognitive decay, due to an available set of motivational abilities or processes (Forstmeier and Maercker 2008; Forstmeier et al. 2012). Although the underlying processes of the relationship between motivational abilities and resilience against cognitive decline have yet to be thoroughly investigated, previous studies examining MR support its protective influence. For instance, studies that applied diversely operationalised MR constructs found cross-sectional and longitudinal evidence for a protective influence of MR against abnormal cognitive decline (i.e. mild cognitive impairment, Alzheimer’s disease) in later life (Forstmeier and Maercker 2008, 2015; Forstmeier et al. 2012; Thoma et al. 2018). Regarding general motivational abilities (e.g. conscientiousness, self-efficacy), research has repeatedly shown that motivational abilities are positively associated with beneficial (health) outcomes in older adults (e.g. Bogg and Roberts 2013). On the basis of this research, combined with the evidence in the MR studies, it may be assumed that the protective influence of MR could apply to both cognitive and other, non-cognitive aspects of SA. Given that the larger project on the basis of which the current study is based upon was planned with the aim to identify early signs of mild cognitive impairment and dementia, the applied battery of instruments did not intent to assess motivational abilities directly, e.g. using behavioural tasks or informant-reports. As such, for the current study, the MR construct was operationalised indirectly via occupation-related motivational activities (i.e. goal orientation and action planning) of the participant’s main (longest) occupation (for a detailed description see Materials and Methods section, *Occupation-related proxy indicators of the CR and MR constructs).* Given that (a higher level of) motivational abilities have previously shown to have a broad beneficial impact, it is expected that MR will be positively related to the general SA factor.

## Materials and methods

### Participants

The current analyses used the same sample as in Kleineidam et al. (2018), which was derived from the AgeCoDe study. The AgeCoDe study is a longitudinal, general practice, registry-based cohort study of older individuals that started between January 2003 and November 2004. Participants were recruited from six German cities (Bonn, Düsseldorf, Hamburg, Leipzig, Mannheim, and Munich). The inclusion criteria were: an age of 75 years or older, absence of dementia according to general practitioner (GP) judgement, and at least one contact with a GP in the previous 12 months. Exclusion criteria were: GP consultations by home visits only, living in a nursing home, severe illness with an anticipated fatal outcome within three months, insufficient knowledge of the German language, deafness or blindness, and inability to provide informed consent. Further details about the recruitment of participants can be found in Luck et al. (2007).

The current study included 2,478 individuals who were interviewed in person at the second follow-up (FU2), which was conducted between 2005 and 2007 and included a more detailed assessment of neuropsychological and psychosocial variables (see Supplementary Material, Figure A, for the flowchart of recruitment and samples). Only cross-sectional data from this (FU2) follow-up was used in this analysis. Between baseline and FU2 assessments, 301 participants died and 548 participants dropped out for other reasons (most frequent reason: refusal to participate further, 87.4%). Participants who dropped out before FU2 were older (79.4 years (3.4) vs. 80.4 years (4.0), *p* < 0.001) and less educated (low education in 60.7% vs. 66.9%, *p* = 0.001). There were no significant differences in gender distribution (*p* = 0.232).

The AgeCoDe study was approved by the Ethics Committee of the Medical Association of Hamburg (file OB/8/02) and the committees of all other participating centres provided local approval for study participation. All participants gave informed consent for participation in the study.

### Operationalisation of different SA constructs

For the operationalisation of SA, the measures and procedure described by Kleineidam et al. (2018) were used. In the current study, measures were assigned to the three facets commonly used to operationalize SA, namely physiological, well-being, and social facets (Cosco et al. 2014). Within each facet, measures were assigned to different response categories and were allocated to different latent factors based on exploratory factor analysis. An overview and scoring rules for the items can be found in Table 1.

**Table 1:** Indicators used for the operationalisation of successful ageing

SA Indicator	Categories	Items
**Physiological facet**		
*Physical and mental health factor*		
Medical conditions with higher mortality risk	0 diseases/1 disease / > 1 disease	Severe diabetes, congestive heart failure, renal disease, liver disease, asthma, or COPD, cancer, dementia
Medical conditions with low mortality risk	Quartiles	Mild diabetes, hypertension, coronary heart disease, myocardial infarction, hyperlipidemia, stroke, osteoarthritis, rheumatoid arthritis
Sensory impairment	No/mild/severe impairment	Self-rated loss in hearing or vision
Impairment in walking	No/mild/severe impairment	
Mental disorder (GP-report)	Presence or absence	Depression, alcohol dependency, other mental disorders
IADL Scale	Gender-specific tertiles	
*Cognitive health factor*		
CERAD word list immediate recall	Quintiles	
CERAD word list delayed recall	Quintiles	
CERAD verbal fluency (animals)	Quintiles	
Clock Drawing Test	Quartiles	
Mini Mental State Examination	Quintiles	
Global Deterioration Scale	Quintiles	
**Social facet**		
*Social activity factor*		
Two items from the social support questionnaire (FSozU K-14)	Agree or disagree	Example items: I know several people I like to go out with There is a group of people that I belong to and that I feel comfortable with
Frequency of: Playing board games Doing social work in the community Taking care of others	Five-point scale	
*Social support factor*		
Twelve items from the social support questionnaire (FSozU K-14)	Agree or disagree	Example items: Experience a lot of sympathy and comfort I have friends/relatives that I can ask to go shopping for me when I am ill
**Well-being facet**		
*Negative affect factor*		
Ten items of the Geriatric Depression Scale	Presence or absence of symptom	- Example items: Do you feel worthless at the moment? Have you abandoned a lot of your interests and activities?
*Positive affect factor*		
Five items of the Geriatric Depression Scale	Presence or absence of symptom	Example items: Are you happy most of the time? Do you feel full of energy?

CERAD = The Consortium to Establish a Registry for Alzheimer’s Disease; COPD = Chronic Obstructive Pulmonary Disease; GP = General Practitioner; FSozU K-14 = Fragebogen zur sozialen Unterstützung: Kurzform (K-14) [Social Support Questionnaire: Short form (K-14)]; IADL = Instrumental Activities of Daily Living

### Measures of the physiological facet

The number of recorded medical diagnoses was reported by the GP using a questionnaire. To rate participants’ freedom from disease, the following health conditions and problems were identified based on the study of Fuchs et al. (2013): Fourteen medical conditions (diabetes, congestive heart failure, renal disease, liver disease, asthma or chronic obstructive pulmonary disease (COPD), cancer, dementia, hypertension, coronary heart disease, myocardial infarction, hyperlipidemia, stroke, osteoarthritis, rheumatoid arthritis) and two health problems (impairments in walking and sensory deficits). Medical conditions were further subdivided into those with a higher one-year mortality risk and those with lower one-year mortality risk (Quan et al. 2011). The GP also reported the presence of any mental health disorder. In addition, self-rated loss of hearing and/or vision, as well as impairments in walking were assessed. The number of independently performed instrumental activities of daily living (IADL) was quantified using the Lawton and Brody scale (Lawton and Brody 1969). A factor measuring physical and mental health was derived from the above medical, functional, and mental health items (‘physical and mental health factor’). Cognition was assessed by applying the Mini Mental State Examination (MMSE, Folstein et al. 1975), as well as the verbal fluency task and the immediate and delayed recall task from the Consortium to Establish a Registry for Alzheimer’s Disease—Neuropsychological Assessment (CERAD-NP, Morris et al. 1989). Furthermore, the Clock Drawing Test (Ihl et al. 2000) and the Global Deterioration Scale (GDS, Reisberg et al. 1982, completed by the interviewer) were administered. A factor indicating cognitive health was constructed from the above cognitive items (‘cognitive health factor’).

### Measures of the social facet

Three items (i.e. playing board games, doing social work in the community, and taking care of others) from a scale assessing cognitive and physical activities in older adults (Verghese et al. 2003) were used to assess social activities. In addition, the 14-item short form of the questionnaire for social support by Fydrich and colleagues (Fydrich et al. 2009) was administered. Based on these 17 items, two factors were created for the social facet: one factor measuring activities in a social group (‘social activity factor’: assessed by the three social activity items and 2 items from the social support questionnaire); and one factor measuring social support (‘social support factor’: assessed by 12 items from the social support questionnaire).

### Measures of the well-being facet

Subjective well-being can be defined as “… the people’s longer-term levels of pleasant affect, lack of unpleasant affect, and life satisfaction “ (Diener 2009, p. 25). In the current study, the well-being facet has been operationalised with the use of the 15 dichotomous items of the short version of the Geriatric Depression Scale (Yesavage and Sheikh, 1986), which were aggregated into two factors indicative of (1) negative affect (‘negative affect factor’), and (2) satisfaction with life (‘positive affect factor’).

### Assessments of potential correlates of SA

#### Sociodemographic variables

Participants were asked to indicate the occupation they had performed for the longest duration during their life. Based on this, socioeconomic status was determined using the *International Socio-Economic Index* (ISEI, Ganzeboom et al. 1992). Years of education were computed according to the reported highest level of schooling and/or professional training. Current marital status was recorded (response options: a) married, b) widowed, c) single, or d) divorced) and participants were divided into two groups (married or widowed versus single or divorced). This approach was chosen to differentiate between individuals that were currently living or had lived in a long-term partnership (married or widowed) and those with no long-term partnerships or with short or interrupted partnerships (single or divorced). The creation of a dichotomous category was necessary to ensure a sufficient number of participants in each category. However, this categorization does not allow for the identification of currently married or widowed participants who only recently entered a partnership (e.g. due to a recent marriage). As such, this categorization bears the potential for measurement error, as it could underestimate the association between SA and marital status.

#### Health behaviours and ApoE-ε4 genotyping

Aspects of health-related behaviours were also assessed. At FU1 (1.5 years before the current study), participants were asked if they had ever smoked on a regular basis. This was taken as a measure of life-time smoking status. Physical and cognitive activities were also assessed using the questions and rating scales originally developed by Verghese and associates (2003). As items from the same scale were used to operationalize social activities for the social facet of the SA constructs (see above), social activity items were excluded when computing the indices for physical and cognitive activities. Example items for cognitive activities include “How often do you read books and newspapers?”, and “How often do you solve crossword puzzles?”. Example items for physical activity include “How often do you go swimming?”, and “How often do you go cycling?”. Items were summed for each category, resulting in a score ranging from 0 to 42 for physical activities and 0–35 for cognitive activities. One point on each scale corresponds to participation in one activity for one day per week. The presence of at least one *ApoE*-ε4 allele was assessed by using standard assessment methods for genotyping, as described in (Luck et al. 2014).

#### Occupation-related proxy indicators of the CR and MR constructs

The influence of midlife psychosocial factors on SA was also assessed. On the basis of participants’ main (longest) occupation, three occupation-related reserve constructs were built. Two CR constructs, i.e. the occupational cognitive requirements score (Pool et al. 2016) and the occupational CR model, were used as indices for occupation-related midlife cognitive activity. The MR construct, which assesses MR-related occupational skills as defined by Forstmeier and Maercker (Forstmeier and Maercker 2008), was used to index occupation-related midlife motivational abilities. In order to build the three reserve constructs, information on job title, duration, and major activities and duties of participant’s former occupation were collected. The longest-held occupation was coded according to the O*NET standard occupational classification (http://www.onetonline.org) by two independent raters. The O*NET is the official occupational classification system of the U.S. Department of Labor and contains a hierarchical structured lexicon of occupations, as well as skills and abilities needed for each occupation. In the case of disagreement between raters, a third rater was consulted and the coding was discussed until a consensus was reached. The initial inter-rater agreement was high at the highest level of aggregation (86%) of the hierarchical O*NET coding system. After coding the occupations of each participant, the reserve indices were constructed by averaging the respective O*NET variables in the case of the OCRS, and by constructing a z-score of the variables in the case of the occupation-related CR and MR. The respective O*NET variables are displayed in supplementary table A (see Supplementary Material, Table A).

### Statistical analysis

A confirmatory factor analysis (CFA) model was constructed with categorical indicators for each facet of the applied SA constructs using the software Mplus (Muthen and Muthén, 1998). This process is described in more detail in (see Kleineidam et al. 2018). A single bifactor model (Reise 2012) was fitted to the data to define the SA constructs which is constituted of three different facets (i.e. a physiological, a social, and a well-being facet). These SA facets are assessed by the items described above (see Fig. 1). A bifactor model imposes a latent structure, in which each item loads on two sets of latent factors: (1) one general factor, which represents the commonalities shared between all facets; and (2) several specific factors, which represent systematic variance that is not shared by all facets or accounted for in the general factor. A bifactor model of SA therefore examines the unity (represented in the general factor), and the diversity (represented in the specific factors) of the facets that collectively compose the SA construct. Any correlations between the facets is captured by the common general SA construct (i.e. the general factor in the bifactor model). Thus, when examining potential correlates and predictors of SA (such as for instance gender or education), an association between correlates of SA and the general factor (SA) indicates that these correlates influence each of the SA facets simultaneously. However, each facet is also characterized by unique aspects that are not shared with the other facets (such as for instance the specific cognitive health factor). Any association between correlates of SA and a specific factor, in addition to the general factor (SA), indicates a stronger or weaker relationship of the correlate with the respective facet, over and above the general factor (SA). Whether the association is stronger or weaker depends on the direction of the effects with the general and specific factor, respectively. If a correlate is associated exclusively with one of the specific factors, but not with the general factor, this indicates that the correlate is not related to the underlying general SA construct and thus does not influence multiple SA facets.

When fitting the bifactor model of SA, only specific factors within each facet were allowed to correlate, with all other factors modelled as uncorrelated. The theta parameterization and WLSMV estimator was used for the CFA model. The metric of the general latent factor was identified by constraining its variance to 1. The metric of the specific factors was determined by constraining the loading on one indicator to be equal to the loading of the general factor on this indicator. This ensured that the general and specific factors were measured on approximately the same scale. No further measurement invariance constraints were included as the associations were compared on a qualitative basis. The model fit was evaluated using comparative fit index (CFI), Tucker-Lewis index (TLI), and root mean square error of approximation (RMSEA). A CFI and TLI > 0.90 and a RMSEA < 0.08 indicated an adequately fitting model (Kline 2015). Loadings of the latent factors of the CFA on the respective factors are displayed in supplementary table B (see Supplementary Material, Table B).

All indicators of the physiological, social, and well-being facets were used as indicators for the *general SA* construct. The specific factors only loaded on the indicators of a specific facet. As described in (Kleineidam et al. 2018), several specific factors were modelled based on the results of exploratory factor analyses. Regarding the measures of the physiological facet, two factors were modelled, representing (a) somatic diseases and impairment in activities of daily living, and (b) cognitive abilities. Similarly, two factors were also modelled for the well-being facet, representing (a) absence of negative affect, and (b) satisfaction with life. Finally, two factors were modelled for the social facet, representing (a) activities and comfort in a social group, and (b) subjective social support and personal familiarity.

A residual correlation between the CERAD-NP immediate and delayed recall was included to account for a common method effect. A cross-loading between the item ‘problems with memory’ from the well-being facet and the cognitive health factor of the physiological facet was also included, as suggested by modification indices, to account for the interrelation of subjectively perceived and objectively quantified cognitive performance.

Missing data were handled using multiple imputations, with twenty imputations, in order to produce unbiased estimates under the ‘missing at random’ assumption. Unstandardized and standardized parameter estimates (i.e. in correlational metric) were reported and average model fit indices were pooled over imputations. The associations between SA and the evaluated correlates were assessed by regressing the global factor and all specific factors on each of the observed associations and correlates. Positive associations with the SA general or specific factors indicate a more favourable ageing course. Separate models were fitted for each association and correlate. Age was controlled for in all analyses. Bonferroni-Holm corrected *p* values were reported to address the potential issue of false positive findings due to multiple testing.

## Results

### Sample characteristics

Table 2 shows the descriptive statistics of the sample (see Table 2). Participants who dropped out between baseline and FU2 (i.e. the visit used in this analysis) were significantly older at baseline (80.5 years (4.1) vs. 79.4 years (3.5); *p* < 0.001) and less educated (34.2% vs. 39.2%, *p* = 0.001) than remaining participants. However, no differences were found in gender distribution (*p* = 0.152).

**Table 2:** Sample characteristics

	Mean (SD)/*n* (%)	Missing *n* (%)
**Socio-demographic variables**		
Age	82.47(3.47)	0 (0.0%)
Female *n* (%)	1635 (66.0%)	0 (0.0%)
Years of education	12.03 (2.26)	0 (0.0%)
SES	44.61 (14.79)	17 (0.9%)
Married or widowed *n* (%)	2200 (88.8%)	5 (0.2%)
**SA indicators**		
No disease with high mortality risk *n* (%)	1627 (65.7%)	141 (5.7%)
Number of diseases with lower mortality risk	1.87 (1.22)	115 (4.6%)
No impairments in hearing and vision *n* (%)	1263 (51.0%)	167 (8.4%)
No mental disorder *n* (%)	1807 (76.5%)	115 (4.6%)
Mini Mental State Examination	27.43 (2.91)	28 (1.1%)
CERAD delayed word recall	5.67 (2.67)	84 (3.4%)
Global Deterioration Scale	1.93 (1.03)	1 (0.0%)
Number of unimpaired activities of daily living	6.68 (1.82)	10 (0.04%)
Number of occasionally performed social activities	0.76 (0.80)	19 (0.8%)
Total score for social support (FSozU K-14)	12.29 (2.53)	71 (2.9%)
Geriatric Depression Scale	2.52 (2.49)	41 (1.7%)
No disease with high mortality risk *n* (%)	1627 (65.7%)	141 (5.7%)
**SA determinants**		
Ever smoked *n* (%)	1140 (46.0%)	21 (0.8%)
Cognitive activities performed (per day per week)	11.2 (5.06)	21 (0.8%)
Physical activities performed (per day per week)	9.96 (6.12)	24 (0.9%)
OCRS	3.21 (0.44)	16 (0.6%)
Occupation-related cognitive reserve	0.00 (0.79)	16 (0.6%)
Occupation-related motivational reserve	-0.00 (0.87)	16 (0.6%)

FSozU K-14 = Fragebogen zur sozialen Unterstützung: Kurzform (K-14) [Social Support Questionnaire: Short form (K-14)]; MMSE = Mini Mental State Examination; *n* = number; OCRS = Occupational cognitive requirements score; SA = Successful ageing; SES = Socio-economic status; SD = Standard deviation

### Associations and correlates of SA

All CFA models showed good fit to the data (RMSEA = 0.026–0.027, CFI = 0.951–0.955, TLI = 0.946–0.950). From previously proposed associations and correlates of SA, the following correlates of the general SA factor were identified: younger age, male gender, higher level of education and SES, marital status (i.e. being married or widowed), as well as physical and cognitive activities (see Table 3 for the full results of the correlates of SA). Besides the associations with the general factor, additional relationships were observed with the factors representing aspects unique to the individual SA facets. Older age was negatively associated with the physical and cognitive health factor, but positively associated with the well-being factors. Male gender showed a strong negative influence on the physical and mental health factor. Education, SES, as well as cognitive and physical activities were positively associated with the cognitive health factor. Education and physical activity were associated with increased negative affect, as indicated by the negative association with the factor ‘absence of negative affect’. In addition, physical activities were negatively associated with the social support factor. Being married or widowed was positively associated with the social support factor in comparison to being single or divorced. Smoking was the only correlate that was not associated with the general factor, but was selectively associated with the physical and mental health factor.

**Table 3:** Results of the prediction of three successful ageing constructs

	General SA factor			Physiological facet						Social facet						Well-being facet					
				Physical and mental health factor			Cognitive health factor			Social support factor			Social activity factor			Positive affect factor			Absence of negative affect factor		
	Est. (SE)	Std. Est	*p*	Est. (SE)	Std. Est	*p*	Est. (SE)	Std. Est	*p*	Est. (SE)	Std. Est	*p*	Est. (SE)	Std. Est	*p*	Est	Std. Est	*p*	Est	Std. Est	*p*
**Previously proposed SA determinates**																					
Age	−0.08 (0.01)	−0.27	** < 0.001**	−0.03 (0.01)	−0.15	** < 0.001**	−0.10 (0.02)	−0.18	** < 0.001**	0.06 (0.02)	0.09	0.567	0.01 (0.01)	0.03	ns	0.07 (0.01)	0.21	**0.001**	0.06 (0.01)	0.28	** < 0.001**
Gender	0.26 (0.06)	0.12	**0.002**	−0.71 (0.19)	−0.48	**0.011**	−0.33 (0.11)	−0.08	0.162	0.35 (0.18)	0.07	ns	−0.16 (0.07)	−0.08	ns	0.15 (0.10)	0.08	ns	−0.07 (0.10)	−0.03	ns
Education	0.07 (0.01)	0.15	**< 0.001**	−0.05 (0.02)	−0.14	0.161	0.16 (0.03)	0.18	** < 0.001**	−0.06 (0.03)	−0.06	ns	0.00 (0.01)	−0.01	ns	−0.02 (0.02)	−0.04	ns	−0.07 (0.02)	−0.18	**0.014**
SES	0.01 (0.00)	0.10	**0.009**	0.00 (0.00)	−0.06	ns	0.02 (0.01)	0.18	** < 0.001**	−0.01 (0.01)	−0.06	ns	0.00 (0.00)	−0.02	ns	0.00 (0.00)	-0.03	ns	0.00 (0.00)	−0.07	ns
Marital status^**a**^	0.29 (0.09)	0.09	**0.049**	−0.30 (0.11)	−0.13	0.312	−0.21 (0.15)	−0.04	ns	1.42 (0.32)	0.18	**0.001**	0.18 (0.09)	0.07	ns	0.01 (0.12)	0.00	ns	−0.06 (0.13)	−0.02	ns
Physical activity	0.06 (0.01)	0.34	** < 0.001**	0.05 (0.02)	0.38	0.141	0.06 (0.02)	0.16	**0.010**	−0.060 (0.01)	−0.16	**0.003**	0.00 (0.01)	0.01	ns	0.00 (0.01)	0.01	ns	−0.04 (0.01)	−0.29	** < 0.001**
Cognitive activity	0.05 (0.01)	0.23	** < 0.001**	0.05 (0.02)	0.29	0.180	0.183 (0.04)	0.36	** < 0.001**	−0.01 (0.01)	−0.03	ns	0.02 (0.01)	0.10	0.831	0.02 (0.01)	0.10	ns	−0.01 (0.01)	−0.08	ns
Ever smoked	0.09 (0.06)	0.04	ns	−0.38 (0.11)	−0.25	**0.049**	−0.16 (0.09)	0.04	ns	−0.32 (0.15)	−0.07	ns	−0.15 (0.07)	−0.09	0.816	−0.01 (0.08)	−0.00	ns	0.00 (0.09)	0.00	ns
**Novel SA determinants**																					
ApoE-ε4-carrier	0.07 (0.07)	0.03	ns	−0.07 (0.06)	−0.04	ns	−0.75 (0.16)	−0.16	** < 0.001**	0.06 (0.19)	0.01	ns	−0.10 (0.07)	−0.05	ns	−0.13 (0.10)	−0.06	ns	−0.24 (0.10)	−0.12	0.805
OCRS	0.25 (0.06)	0.10	**0.009**	−0.28 (0.09)	−0.17	0.099	0.42 (0.13)	0.10	**0.043**	−0.0 (0.18)	−0.01	ns	−0.08 (0.07)	−0.04	ns	0.03 (0.09)	0.02	ns	−0.02 (0.10)	−0.01	ns
Occupation-related cognitive reserve	0.12 (0.04)	0.09	0.075	−0.15 (0.05)	−0.16	0.141	0.18 (0.07)	0.07	0.261	0.03 (0.10)	0.01	ns	0.00 (0.04)	−0.00	ns	0.04 (0.06)	0.03	ns	−0.07 (0.06)	−0.07	ns
Occupation-related motivationalreserve	0.11 (0.03)	0.09	0.062	−0.14 (0.05)	−0.17	0.099	0.21 (0.06)	0.09	0.053	−0.11 (0.09)	−0.04	ns	−0.03 (0.03)	−0.03	ns	0.00 (0.05)	0.00	ns	−0.02 (0.05)	−0.02	ns

^a^ = Reference category is single or divorced; Est = Unstandardized estimate from the confirmatory factor analysis with WLSMV estimator; Std. Est. = Standardized estimate in correlational metric; SA = Successful ageing; SE = Standard error; *p* = *p*-value adjusted for multiple testing using Bonferroni-Holm correction; ns = Not significant, i.e. *p* > .999; SES = Socio-economic status; ISEI = International Socio Economic Index; OCRS = Occupational cognitive requirements score; significant results are highlighted in bold

Among the novel determinants of SA, only the OCRS construct was associated with the general SA factor, although the occupation-related MR construct showed trend-level associations with the general SA factor. The OCRS and MR constructs showed an additional significant positive association with the cognitive health factor. Carrying the *APOE-ε4* allele was not associated with the general SA factor, but was negatively associated with the cognitive health factor.

## Discussion

Successful ageing is an appealing and frequently employed research construct involving different aspects of biopsychosocial functioning (Kleineidam et al. 2018). It was unclear in the literature whether previously identified associations and correlates of SA simultaneously affected all components of the multifaceted SA construct, or whether the associations and correlations derived solely from effects on specific, selective facets of the holistic SA construct. It was therefore the aim of the current study to identify the associations and correlates of SA that exert a broad and simultaneous effect on all components of SA. Further investigations into the causal impact of these factors would be particularly important with regard to public health recommendations, as targeted intervention for these general factors could maximize the impact on the ageing course as a whole, compared to interventions for specific factors that influence only single aspects of SA.

Previous research on SA determinants rarely investigated the influence of SA associations and correlates across several different facets of the multifaceted SA construct. The bifactor modelling approach applied in this study allowed for the dissection of the associations with SA, which offered a novel way to rank the importance of SA associations and correlates. In addition, this approach presented an explanation for the inconsistencies in findings of previous studies, which were induced by the application of different operational definitions of SA. For the majority of the SA associations and correlates that were previously proposed in the literature, the current study demonstrated an association with the general SA factor, indicating the expected broad effect on all SA facets. However, additional relationships with the unique, facet-specific factors were also identified, pointing towards stronger associations between the correlates and some SA facets, over and above the relationship with the shared underpinning of all SA components.

In line with previous studies, younger age showed one of the strongest associations among all examined SA correlates (e.g. Depp and Jeste 2006; Ng et al. 2009). However, younger age showed a disproportionately strong positive association with physical and cognitive health; with a less pronounced association between younger age and well-being, as indicated by the negative association with the specific factors beyond the general SA factor. Ageing per se might therefore exert its largest effect on physiological health, but this may not translate into the subjective experience of age-related deterioration to the same extent.

In the current study, male gender was observed to be positively associated with the general SA factor, but also showed a strong negative association with the physical and mental health factor, which seemed to exceed the effect on the general SA factor in magnitude. This implies that the effect of male gender may depend on the operationalisation of SA (McLaughlin et al. 2010, 2012). Biomedically-oriented definitions might predominantly identify the strong negative association of male gender and physical health, while broader definitions may pick up on the relationship with the more subjective aspects of SA. This may explain the controversy in the literature, in which the role of gender has been extensively discussed (e.g. Britton et al. 2008; Pruchno, Wilson-Genderson and Cartwright 2010a, b).

High SES was also identified as a correlate of the holistic SA construct. In addition, SES showed a particularly strong effect on cognitive health. These results indicate that an improvement in this variable may result in a more favourable ageing course in general, and preserved cognitive abilities in particular. These findings corroborate previous reports on the influence of SES on ageing (Britton et al. 2008; McLaughlin et al. 2010; Ng et al. 2009). Pronounced associations were observed for higher education and the general SA factor, as well as the specific cognitive health factor. However, a negative association with the ‘absence of negative affect’ factor indicated a weaker effect (compared to other SA facets) of education on aspects of well-being. In the current study, negative affect was operationalised with depression-related, negative affect items. It may be that this operationalisation led to this particular finding, i.e. that education does not seem to be a strong determinant of the presence or absence of negative affect.

Furthermore, being married or widowed was shown to be related to the general SA factor. In addition to this association, a relationship was found with the factor ‘social activity’, which highlights the importance of close, intimate relationships in midlife for SA. While this association has not been detected in many biomedically-oriented models, the current findings are supported by previous research that included subjective SA measures, suggesting that there is an association between social aspects and SA.

With regard to behaviour-related variables, physical activity was identified as the strongest correlate of SA, which is in line with previous reports (Hodge et al. 2013; Ng et al. 2005, 2009; Vaillant and Mukamal 2001). Although a detailed examination is required for the causal role of physical activity in SA, the current findings point towards the potential of this modifiable factor for SA interventions. In addition, the results show that there was a negative association between physical activity and the factors ‘social support’ and ‘absence of negative affect’, suggesting that the effect of physical activity is less pronounced with respect to subjective (as opposed to biomedical) aspects of SA.

No association was found between smoking and the general SA factor, but an effect was observed for the specific factor of physical health. Non-smoking seems to be uniquely beneficial for physical health and may not be generalized to other SA facets. Although smoking has been identified as a SA correlate by multiple, predominantly biomedically-oriented models (Britton et al. 2008; Hodge et al. 2005, 2013; Vaillant and Mukamal 2001); the current findings call into question the usefulness of smoking modification interventions to promote SA, at least when targeting the well-being and social function of participants.

Carrying the *APOE-ε4* allele was negatively associated with the cognitive health factor; no association was found with the general SA factor. That the *APOE-ε4* allele is important in predicting pathological cognitive decline was shown in the Nun study, where the *APOE-ε4* allele was found to be a relevant predictor in the transition from unimpaired to mildly impaired cognitive abilities (Tyas et al. 2007). As such, this biological index may be more relevant in the understanding of the pathological cognitive as opposed to the salutogenic ageing course.

Moreover, (current) cognitive activity (i.e. cognitive activities performed per day per week; Verghese et al. 2003) was identified as a significant correlate of SA. This finding corresponds to a study by Menec (2003), which reported that activities, such as reading, writing, or music/art/theatre, are prospectively associated with SA-related measures. In line with this, the current study found that higher scores of the reserve construct OCRS (indicating higher occupational cognitive requirements in midlife) were associated with higher SA. This is, to the best of the authors’ knowledge, a novel finding. Combined with the observation of associations between SA and higher education, as well as (current) cognitive activities, this finding emphasizes the importance of lifelong cognitive stimulation and active engagement in cognitive processes for SA. Interestingly, a much less pronounced, non-significant (after correction for multiple testing) association was observed between SA and the occupation-related MR construct. The MR construct focused more on motivational abilities that are required for or are related to the pursuit of work-related cognitive activities, while the OCRS assessed the occupational cognitive requirements that were engaged during the participants’ longest-held jobs. This suggests that the strongest associations with SA are related to the actual practice of cognitive activities, and to a lesser extent, motivational abilities and skills. Further research is needed to investigate whether occupational cognitive requirements in midlife, education, and cognitive activities in later life are related to SA by the same mechanisms proposed for their association with dementia. Further insights are particularly important as large, multi-domain prevention trials for Alzheimer’s disease are currently underway (The 2017). If such multi-faceted interventions can also promote a favourable ageing course in general, as suggested by the current findings, the value and potential outcomes for these trials could be broader than currently expected. However, the lack of association between the *ApoE-ε4* allele and SA in this study suggests that not all interventions directed at Alzheimer’s disease would also be beneficial for SA. Notably, physical exercise and cognitive activity throughout the life course, the strongest correlates of SA in the current study, should be in the focus of public health policies.

A major strength of the current study was the use of a previously validated SA definition (Kleineidam et al. 2018) within one large sample. The use of bifactor modelling was another strength, which allowed distinctions to be made for the first time between shared associations of SA correlates with all SA facets, or confined relationships with a specific SA aspect. In addition, in contrast to the majority of previous research on the associations and correlates of SA, the current study did not rely solely on self-reports, but also included health measures assessed by the GP.

This study comes with some limitations: Due to the lack of longitudinal analysis, it cannot be concluded form the data whether the identified associations and correlates are mechanistically causal. However, as the applied indices also indexed past behaviour and experiences in adolescence, young, and middle adulthood (e.g. education, SES, marital status), the chronological sequence of events is evident. As the larger study was planned and conducted with the aim of identifying early signs of mild cognitive impairment and dementia, the applied methodology, including the psychometric instruments, are biased toward the detection of neuropathological development. As such, the battery of instruments did not include instruments related to psychological resources (e.g. resilience, conscientiousness), well-being, or the use of technology. The latter could have been used as a novel determinant for SA (e.g. Khosravi et al. 2016). The lack of the (direct) assessment of various psychological resources and well-being had an impact on the operationalisation of the constructs and facets applied. For instance, the current study operationalised the MR construct indirectly via occupation-related motivational activities (i.e., goal orientation and action planning) of the participant’s main (longest) occupation following the procedure by Forstmeier and Maercker (2008). This method has previously found to be useful in assessing MR in the absence of directly measured motivational abilities (Forstmeier et al. 2012). With respect to the operationalisation of the various facets, it is in particular the well-being facet that is comparatively weak. As we used a screening questionnaire for depression, our well-being operationalisation most closely focuses on the lack of unpleasant affect. Future studies are advised to apply a more elaborate well-being facet by measuring various aspects of the complex well-being construct. Given that race and cultural background have also been shown to exert an impact on SA, the lack of diversity in the current sample hindered the examination of related research questions. In addition, the sample was rather old and may therefore have been affected by a selective mortality bias (Peel et al. 2005). Furthermore, individuals who received GP consultations by home visits only or who lived it nursing institutions were not included in the study. This limits the generalizability of the findings to the general public, due to a selection bias towards more mobile and healthy older adults in the investigated age segment. Further, the (occupation-related) MR construct constitutes a relatively recent developed construct. As such, there is only a limited understanding of the underlying processes involved in its protective impact, which may be complexly intertwined with protective influences stemming (predominantly) from cognitive, physical, or social activities. Finally, items from the physical and cognitive activity scale developed by Verghese and colleagues (2003) were also used to define the social facet of the SA factor. Common method bias may have influenced the association of these correlates with the SA constructs.

This study demonstrated that most, but not all, previously considered associations and correlates of SA are simultaneously related to all aspects of the holistic SA concept, and also that some correlates show only specific effects on certain SA aspects. Age, education, as well as physical and cognitive activity appear to be particularly robust associations and correlates for SA. Novel associations were also identified between SA and occupational cognitive requirements in midlife, which is considered to be proxy indicator of CR. Strikingly, the most consistently identified correlates were indicators of a lifelong active and vigorous lifestyle, which has previously been associated with reduced risk for dementia (Stern 2012). This stresses the need for further investigation into whether or not these activities causally induce favourable ageing trajectories in general. According to the current results, occupation-related cognitive and psychosocial activities in midlife could be important contributors to SA. Associations and correlates other than those reported above appear to be less robust and could therefore vary as a function of the emphasis of the SA construct. Future research on SA should be aware that the emphasis of an applied SA construct has an influence on which associations and correlates will be identified, and that bifactor modelling could be a useful strategy to identify variables that have a broad impact on all facets of SA.

## Electronic supplementary material

Below is the link to the electronic supplementary material.

Supplementary file1 (DOCX 80 kb)
