# Supplementary material for: Associations and correlates of general versus specific successful ageing components
Source: Eur J Ageing. 2020 Dec 12;18(4):549–63. doi: 10.1007/s10433-020-00593-4 (PMC8563891; doi:10.1007/s10433-020-00593-4)
Supplement: Supplementary file 1 — Supplementary file1 (DOCX 80 kb) [file 10433_2020_593_MOESM1_ESM.docx]

**Supplementary Material**

**Figure A**

Randomly selected sample size

(*n* = 6,619)

**Excluded individuals (*n* = 3,292)**

- Contact failed: 1,517 (22.9%)

- Refused participation: 1,775 (26.8%)

- Main reasons for refusal:

- No interest in the study (58%)

- Felt too weak to participate (13%)

- Lack of time (12%)

Participants: AgeCoDe baseline sample

(*n* = 3,327)

**Drop-outs (*n* = 507, 15.3%)**

- Died: 143 (4.3%)

- Refused: 311 (9.3%)

- Other: 53 (1.6%)

**18 months interval**

In-person interview at

follow-up 1

(*n* = 2,820, 84.8%)

**Drop-outs (n = 342, 10.2%)**

- Died: 158 (4.7%)

- Refused: 178 (5.4%)

- Other: 6 (0.5%)

**18 months interval**

**Sample used for analysis**

In-person interview at

follow-up 2

(*n* = 2,478, 74.5%)

**Table A.** O*Net variables used for the psychosocial reserve models.

| O*Net variable | Description |
| --- | --- |
| **Occupational cognitive requirements score (OCRS)** | |
| 4.A.2.b.6 | Organizing, Planning, and Prioritizing Work |
| 4.A.2.a.1 | Judging the Qualities of Things, Services, or People |
| 4.A.2.a.3 | Evaluating Information to Determine Compliance with Standards |
| 4.A.2.a.2 | Processing Information |
| 4.A.2.a.4 | Analyzing Data or Information |
| 4.A.2.b.1 | Making Decisions and Solving Problems |
| 4.A.2.b.2 | Thinking Creatively |
| 4.A.2.b.3 | Updating and Using Relevant Knowledge |
| 4.A.2.b.4 | Developing Objectives and Strategies |
| 4.A.2.b.5 | Scheduling Work and Activities |
| **Occupation-related cognitive reserve (CR)** | |
| 1.A.1.g.1 | Ability to concentrate on a task over a period of time without being distracted |
| 1.A.1.b.3 | Ability to tell when something is wrong or is likely to go wrong |
| 2.A.2.d | Assessing performance of yourself, other individuals, or organizations to make improvements |
| 2.B.1.a | Being aware of others’ reactions and understanding why they react as they do |
| **Occupation-related motivational reserve (MR)** | |
| 4.A.2.b.6 | Organizing, Planning, and Prioritizing Work |
| 4.A.1.b.3 | Determining time, costs, resources, or materials needed to perform a work activity |

**Table B**

|  | General SA  factor | | Physical and mental  health factor | | Cognitive  health factor | | Social support  factor | | Social activity  factor | | Positive affect factor | | Absence of  negative affect factor | |
| --- | --- | --- | --- | --- | --- | --- | --- | --- | --- | --- | --- | --- | --- | --- |
|  | Est. | SE | Est. | SE | Est. | SE | Est. | SE | Est. | SE | Est. | SE | Est. | SE |
| Impairments  in walking | -0.66 | 0.04 | -0.48 | 0.14 | - | - | - | - | - | - | - | - | - | - |
| Sensory  deficits | -0.22 | 0.03 | -0.22 | 0.03 | - | - | - | - | - | - | - | - | - | - |
| Medical condition  with high mortality risk | -0.21 | 0.04 | -0.60 | 0.19 | - | - | - | - | - | - | - | - | - | - |
| Medical condition  with low mortality risk | -0.09 | 0.03 | -0.24 | 0.09 | - | - | - | - | - | - | - | - | - | - |
| Mental disorder | -0.52 | 0.04 | -0.24 | 0.09 | - | - | - | - | - | - | - | - | - | - |
| IADL | 0.18 | 0.05 | 1.65 | 0.55 | - | - | - | - | - | - | - | - | - | - |
| Delayed  memory recall | 0.38 | 0.04 | - | - | 0.55 | 0.08 | - | - | - | - | - | - | - | - |
| MMSE | 0.23 | 0.04 | - | - | 0.54 | 0.08 | - | - | - | - | - | - | - | - |
| Immediate  memory recall | 0.45 | 0.04 | - | - | 0.54 | 0.08 | - | - | - | - | - | - | - | - |
| Clock Drawing  Test | 0.28 | 0.04 | - | - | 0.28 | 0.04 | - | - | - | - | - | - | - | - |
| GDS | 0.75 | 0.07 | - | - | 0.94 | 0.15 | - | - | - | - | - | - | - | - |
| Verbal fluency | 0.38 | 0.04 | - | - | 0.40 | 0.06 | - | - | - | - | - | - | - | - |
| Support  when moving | 0.38 | 0.07 | - | - | - | - | 0.34 | 0.06 | - | - | - | - | - | - |
| Unconditionally  accepted by others | 0.67 | 0.11 | - | - | - | - | 0.39 | 0.08 | - | - | - | - | - | - |
| Comfort | 0.57 | 0.07 | - | - | - | - | 0.49 | 0.08 | - | - | - | - | - | - |
| Rely on  support | 0.52 | 0.10 | - | - | - | - | 0.57 | 0.10 | - | - | - | - | - | - |
| Borrow things | 0.40 | 0.05 | - | - | - | - | 0.28 | 0.05 | - | - | - | - | - | - |
| Persons  who listen | 0.68 | 0.08 | - | - | - | - | 0.53 | 0.09 | - | - | - | - | - | - |
| Hug | 0.62 | 0.07 | - | - | - | - | 0.46 | 0.08 | - | - | - | - | - | - |
| Support  when ill | 0.46 | 0.09 | - | - | - | - | 0.50 | 0.09 | - | - | - | - | - | - |
| Support  when sad | 0.73 | 0.08 | - | - | - | - | 0.64 | 0.11 | - | - | - | - | - | - |
| Share  suffering | 0.79 | 0.11 | - | - | - | - | 0.68 | 0.12 | - | - | - | - | - | - |
| Being boisterous | 0.72 | 0.06 | - | - | - | - | 0.36 | 0.06 | - | - | - | - | - | - |
| Closeness | 0.48 | 0.07 | - | - | - | - | 0.48 | 0.07 | - | - | - | - | - | - |
| Activities together | 1.13 | 0.13 | - | - | - | - | - | - | 1.84 | 0.47 | - | - | - | - |
| Feel good in a group | 1.00 | 0.10 | - | - | - | - | - | - | 1.63 | 0.40 | - | - | - | - |
| Care for  grandchildren | 0.28 | 0.04 | - | - | - | - | - | - | 0.06 | 0.07 | - | - | - | - |
| Playing  board games | 0.28 | 0.03 | - | - | - | - | - | - | 0.28 | 0.03 | - | - | - | - |
| Social work  in the community | 0.33 | 0.04 | - | - | - | - | - | - | 0.11 | 0.06 | - | - | - | - |
| Satisfaction  with life* | -1.35 | 0.19 | - | - | - | - | - | - | - | - | -1.28 | 0.29 | - | - |
| In good spirit* | -1.01 | 0.11 | - | - | - | - | - | - | - | - | -1.03 | 0.21 | - | - |
| Happy* | -0.91 | 0.08 | - | - | - | - | - | - | - | - | -0.91 | 0.08 | - | - |
| Wonderful  to be alive* | -0.74 | 0.06 | - | - | - | - | - | - | - | - | -0.43 | 0.10 | - | - |
| Full of energy* | -0.76 | 0.05 | - | - | - | - | - | - | - | - | -0.16 | 0.08 | - | - |
| Problems  with memory | -0.41 | 0.08 | - | - | -0.34 | 0.07 | - | - | - | - | - | - | -0.63 | 0.18 |
| Dropped activities | -0.90 | 0.06 | - | - | - | - | - | - | - | - | - | - | -0.08 | 0.08 |
| Life is empty | -1.03 | 0.10 | - | - | - | - | - | - | - | - | - | - | -0.86 | 0.17 |
| Often bored | -0.61 | 0.07 | - | - | - | - | - | - | - | - | - | - | -0.48 | 0.12 |
| Something  bad will happen | -0.27 | 0.04 | - | - | - | - | - | - | - | - | - | - | -0.42 | 0.10 |
| Helpless | -0.96 | 0.08 | - | - | - | - | - | - | - | - | - | - | -0.47 | 0.10 |
| Avoiding social  gatherings | -0.51 | 0.04 | - | - | - | - | - | - | - | - | - | - | 0.12 | 0.08 |
| Worthless | -1.13 | 0.13 | - | - | - | - | - | - | - | - | - | - | -1.13 | 0.13 |
| Hopeless | -1.12 | 0.12 | - | - | - | - | - | - | - | - | - | - | -0.60 | 0.12 |
| Most people are  better off | -0.54 | 0.06 | - | - | - | - | - | - | - | - | - | - | -0.25 | 0.08 |

**Note**. *Item has been reversed, i.e. a score of one (as opposed to zero) indicates rejection of this item. IADL: instrumental activities of daily living. MMSE: Mini Mental State Examination.
